# Adenosine Receptor A1-A2a Heteromers Regulate EAAT2 Expression and Glutamate Uptake via YY1-Induced Repression of PPAR*γ* Transcription

**DOI:** 10.1155/2020/2410264

**Published:** 2020-03-06

**Authors:** Xianhua Hou, Yuan Li, Yuanyuan Huang, Huan Zhao, Li Gui

**Affiliations:** Department of Neurology, Southwest Hospital, Third Military Medical University (Army Medical University), Chongqing 400038, China

## Abstract

Adenosine receptors A1 (A1AR) and A2a (A2aAR) play an important role in regulating glutamate uptake to avoid glutamate accumulation that causes excitotoxicity in the brain; however, the precise mechanism of the effects of A1AR and A2aAR is unclear. Herein, we report that expression of the A1AR protein in the astrocyte membrane and the level of intracellular glutamate were decreased, while expression of the A2aR protein was elevated in cells exposed to oxygen-glucose deprivation (OGD) conditions. Coimmunoprecipitation (Co-IP) experiments showed that A1AR interacts with A2aAR under OGD conditions. The activation of A1AR and inactivation of A2aAR by 2-chloro-N6-cyclopentyladenosine (CCPA) and SCH58251, respectively, partly reversed OGD-mediated glutamate uptake dysfunction, elevated EAAT2, and PPAR*γ* protein levels, and suppressed the expression of Ying Yang 1 (YY1). Both the silencing of YY1 and the activation of PPAR*γ* upregulated EAAT2 expression. Moreover, YY1 silencing elevated the PPAR*γ* level under both normal and OGD conditions. Histone deacetylase (HDAC)1 was found to interact with YY1, and HDAC1 silencing improved PPAR*γ* promoter activity. Taken together, our findings suggest that A1AR-A2aAR heteromers regulate EAAT2 expression and glutamate uptake through the YY1-mediated recruitment of HDAC1 to the PPAR*γ* promoter region.

## 1. Introduction

Ischemic stroke, the most common subtype of stroke, seriously threatens public health in China, with up to 2.5 million new stroke cases reported every year and high rates of mortality [1, 2]. The exposure of astrocytes to oxygen-glucose deprivation (OGD) conditions induces dysfunction of glutamate clearance and inflammatory mediator release, resulting in excitotoxic neuronal death during the ischemic process [3–7]. Therefore, astrocytes, the most abundant glial cell type, play a crucial role in the pathological process of ischemic stroke [8], and the restoration of glutamate clearance function in astrocytes is a therapeutic approach for neuroprotection in ischemic stroke [9].

The role of rapid extracellular glutamate clearance in astrocytes is dependent on excitatory amino acid transporters (EAATs), also known as glutamate transporters (GluTs) [10, 11]. The EAAT family contains five subtypes: EAAT1, EAAT2, EAAT3, EAAT4, and EAAT5 [12]. Studies have revealed that EAAT1 and EAAT2 are predominantly expressed in astrocytes and that EAAT3, EAAT4, and EAAT5 are located in neurons [13, 14]. In particular, EAAT2, the most abundant GluT in the brain [15], plays a major role in the uptake of extracellular glutamate [16–18]. Several studies have reported that the expression of EAAT2 is suppressed in brain tissue suffering from neurological disorders, including ischemia, manganism, and Alzheimer's disease (AD) [10]. Further studies reported that EAAT2/GLT1 expression is regulated at the transcriptional and translational levels through a complex mechanism that involves factors such as PI3K-Akt [19], NF-*κ*B [20], and the epigenetic modifiers histone deacetylase (HDAC) I and II [21]. However, the mechanism of EAAT2 regulation has not been fully clarified.

Adenosine receptor (AR), a member of the G protein-coupled receptor (GPCR) superfamily, consists of four AR subtypes: adenosine receptor A1 (A1AR), adenosine receptor A2a (A2aAR), A2bAR, and A3AR [22]. In particular, A1AR and A2aAR are highly expressed throughout brain tissue and have a high affinity for adenosine [23, 24]. Adenosine binds A1AR and A2aAR and then initiates several biological processes, such as the regulation of glutamate release [25, 26]. Researchers found that A1AR and A2aAR are coexpressed at 61% of presynaptic terminals from cortical glutamatergic neurons in the striatum, caudate nucleus, and hippocampus [27, 28]. Recently, Cristovao-Ferreira et al. demonstrated that A1AR and A2aAR colocalized at the cell surface [28–30]. Further study showed that A1AR could interact with A2aAR to form A1AR-A2aAR heteromers. The activation of A2aAR can uniquely inhibit the A1AR-mediated biological response [31]. In ischemic stroke, the activation of A2aAR by a high concentration of adenosine elevates the intracellular glutamate level and exacerbates neuronal injury [32, 33], whereas adenosine- or pharmaceutical agonist-induced activation of A1AR has been shown to play a protective role under ischemia [34, 35]; A1AR activation reduced glutamate release and promoted ischemic tolerance [36, 37]. However, whether A1AR closely interacts with A2aAR to form A1AR-A2aAR heteromers and the role of A1AR-A2aAR heteromers in ischemic stroke are largely unknown.

Given that a major function of astrocytes in the regulation of glutamate clearance, the first aim of the present work was to clarify whether OGD induces A1AR-A2aAR heteromer formation and modulates glutamate uptake in astrocytes. In addition, we explored the novel mechanism by which A1AR-A2aAR heteromers regulate EAAT2 expression and glutamate uptake in astrocytes.

## 2. Method and Materials

### 2.1. Materials and Reagents

DMEM (with high glucose or no glucose), Opti-MEM™-reduced serum medium (Opti-MEM), fetal bovine serum (FBS), and trypsin-EDTA (0.25%) were obtained from Gibco (Shanghai, China). Anti-A1AR antibody, A2aAR, GFAP, S100*β*, anti-PPAR*γ* antibody, a glutamate assay kit, and 2-chloro-N6-cyclopentyladenosine (CCPA) were purchased from Abcam, Inc. (Shanghai, China). Anti-EAAT2 antibody, anti-*α*/*β*-tubulin antibody, anti-*β*-actin antibody, and anti-Ying Yang 1 (YY1) antibody were purchased from Cell Signaling Technology, Inc. Protein A/G Plus agarose was purchased from Santa Cruz Biotechnology, Inc. (Shanghai, China). CCPA, SCH58261, and CGS21680 were purchased from Selleck, Inc. (Shanghai, China). HRP-goat anti-rabbit IgG (H+L), HRP-goat anti-mouse IgG (H+L), Alexa Fluor® 488-goat anti-rabbit IgG (H+L), and Alexa Fluor® 594-goat anti-rabbit IgG (H+L) were ordered from Jackson ImmunoResearch, Inc. (Philadelphia, USA). GoTaq® qPCR Master Mix, a Luciferase Reporter Gene Assay Kit, GoScript™ RT Mix, and FuGENE® HD Transfection Reagent were purchased from Promega Biotech Co., Ltd. (Beijing, China). T-PER™ Tissue Protein Extraction Reagent and the Mem-PER™ Plus Kit were purchased from Thermo Fisher Co., Ltd. (Shanghai, China). Immobilon Western Chemiluminescence HRP Substrate (ECL kit) was purchased from Merck Millipore Co., Ltd. (Shanghai, China). Polyvinylidene fluoride (PVDF) membranes were purchased from GE Co., Ltd. (Germany). TRIzol reagent and other reagents were purchased and used as received from Sigma-Aldrich (Shanghai, China). The details of materials and reagents are showed in the supplement file ([Supplementary-material supplementary-material-1]).

### 2.2. Mouse Primary Astrocyte Culture and OGD Treatment

Primary cortical astrocytes were isolated and cultured according to a previous report [38]. Briefly, mouse pups were sacrificed by decapitation using scissors. Cortical hemispheres were isolated with fine forceps and then cut into small pieces for trypsinization in a water bath at 37°C for 30 min. Cortex tissue pieces were dissociated into a single-cell suspension and cultured in culture flasks. After culture for 7-8 days, microglia and oligodendrocyte precursor cells were removed by shaking. Astrocytes were used for further study after their culture for an additional 6 days.

For OGD treatment, the culture medium was removed, and astrocytes were washed twice with PBS. Glucose-free DMEM was added to the culture flask, and the astrocytes were exposed to 1% O_2_ for 6 h.

### 2.3. Immunofluorescence Assay

Primary astrocytes were fixed with 4% formaldehyde for 15 min and washed with PBS. These samples were blocked with 5% normal goat serum for 1 h at RT and incubated with anti-GFAP or anti-S100B antibody overnight at 4°C. After being washed three times with PBS, the samples were incubated with Alexa Fluor® 488-goat anti-rabbit IgG (H+L) or Alexa Fluor® 594-goat anti-rabbit IgG (H+L) for 1 h at RT. The nuclei were stained with diamidino-phenylindole (DAPI).

### 2.4. Coimmunoprecipitation (Co-IP) and Western Blotting

Co-IP and western blotting were performed according to a previous study [39]. Briefly, the treated cells were harvested and then centrifuged at 14,000 × g and 4°C for 15 min. The supernatants were then transferred into new tubes and incubated with anti-A1AR antibody (1 : 50) or anti-YY1 antibody (1 : 50) overnight with protein A/G agarose beads for 2 h at 4°C. The solutions were then evaluated by western blotting.

### 2.5. Glutamate Uptake Assay

For the glutamate uptake assay, the treated astrocyte medium was discarded, and the cells were washed with D-Hank's solution. The cells were preincubated with 300 nM glutamate for 7 min, and glutamate uptake was terminated with ice-cold Hank's solution, followed by immediate analysis using a glutamate assay kit [40, 41]. The level of intracellular glutamate was analyzed using a glutamate assay kit (colorimetric) according to the manufacturer's instructions. Briefly, the treated cells and medium were collected, and the cells were resuspended in assay buffer. These samples were incubated for 30 min on ice and centrifuged for 5 min at 4°C. The supernatant was collected, and the reaction mixture was added and incubated with the samples. The absorbance values of the samples were measured at 450 nm.

### 2.6. EYFP-A1AR Plasmid, A2aAR siRNA, YY1 siRNA, and HDAC1 siRNA Transfection

pcDNA3.1/opto-a1AR-EYFP was a gift from Karl Deisseroth (Addgene plasmid #20947). A2aAR and YY1 siRNA were purchased from GenePharma (Shanghai, China). The sequence of A2aAR siRNA was 5′-AAACTTCTTCGTGGTATCTCT-3′ [42]. The sequence of YY1 siRNA was 5′-TTGTTCAATGTAGTCGTCG-3′, and the HDAC1 siRNA sequence was 5′-GTTGGAAGAGTTCTTCGGG-3′. Astrocytes were cultured on 6-well dishes, and 2 *μ*g of plasmid or 200 nM siRNA was used to transfect the astrocytes using FuGENE® HD transfection reagent for 48 h. Treated cells were used for further study.

### 2.7. Luciferase Assay

The promoter region of PPAR*γ* was synthesized and inserted into the pXPG plasmid, which was a gift from Peter Cockerill (Addgene plasmid #71248). This plasmid and a pRL Renilla luciferase control reporter vector were cotransfected into 293 T cells. Luciferase assays were carried out according to the manufacturer's protocol.

### 2.8. Statistical Analysis

The data from at least three independent experiments are expressed as the mean ± standard error (S.E.). One-way analysis of variance (ANOVA) and Student's *t*-test were carried out. *p* < 0.05 indicated statistically significant differences.

## 3. Results

### 3.1. OGD Regulated A1AR and A2aAR Expressions

**Figure 1:**
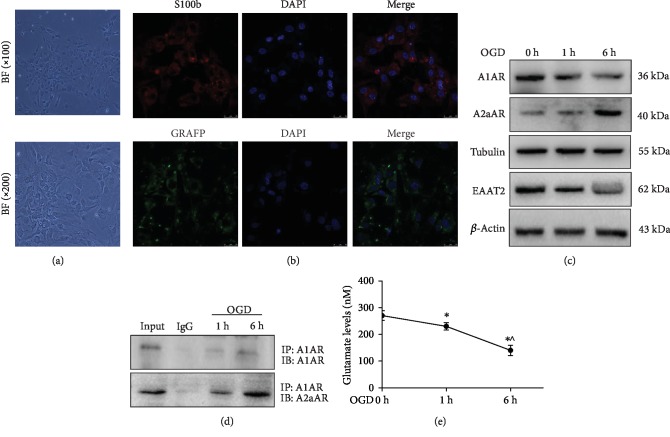
OGD promoted the interaction of A1AR with A2aAR in astrocytes. (a) Normal primary astrocyte morphology was observed by phase-contrast microscopy and is shown in the upper (×100) and lower (×200) panels. (b) Astrocytes were identified by immunofluorescence assay. The positive expression of the S100b (red) and GRAFP (green) antigens in astrocytes was determined by immunofluorescence (×200). Nuclei were stained with DAPI (blue). Astrocytes were exposed to OGD conditions for the indicated times. (c) The protein expression of A1AR, A2aAR, and EAAT2 was evaluated by western blotting. (d) Co-IP assays were used to evaluate the interaction between A1AR and A2aAR. (e) The intracellular glutamate levels were detected using a glutamate assay kit. The data represent the means ± S.E. *n* = 3. ^∗^*p* < 0.05 vs. the 0 h group and ^^^*p* < 0.05 vs. the 1 h group. The data represent as the means ± S.E. (*n* = 3).

Astrocytes play an important role in taking up excess glutamate to avoid excitotoxicity to neurons [11]. Herein, we isolated and cultured mouse primary cortical astrocytes (Figure 1(a)). Immunostaining showed that S100B and GFAP, which are astrocyte markers, were strongly expressed in the cytoplasm of these cells (Figure 1(b)). OGD can be used to develop an in vitro model of brain ischemia. In our study, astrocytes were under OGD for 1 h and 6 h. Western blotting showed that the expression of A1AR on the cell surface was decreased after OGD treatment, whereas A2aAR protein levels were elevated. Furthermore, expression of the EAAT2 protein was also decreased after cells exposed to OGD (Figure 1(c)). Interestingly, the Co-IP assay showed that OGD promoted the interaction between A1AR and A2aAR (Figure 1(d)). Additionally, the intracellular glutamate level was significantly decreased after cells were subjected to OGD for 1 and 6 h compared to 0 h (*p* < 0.05) (Figure 1(e)). These data suggest that A1AR interacts with A2aAR to form an A1AR-A2aAR heterodimer involved in the regulation of EAAT2 expression and glutamate uptake.

### 3.2. A1AR and A2aAR Regulated the Expression of EAAT2 and Glutamate Uptake

**Figure 2:**
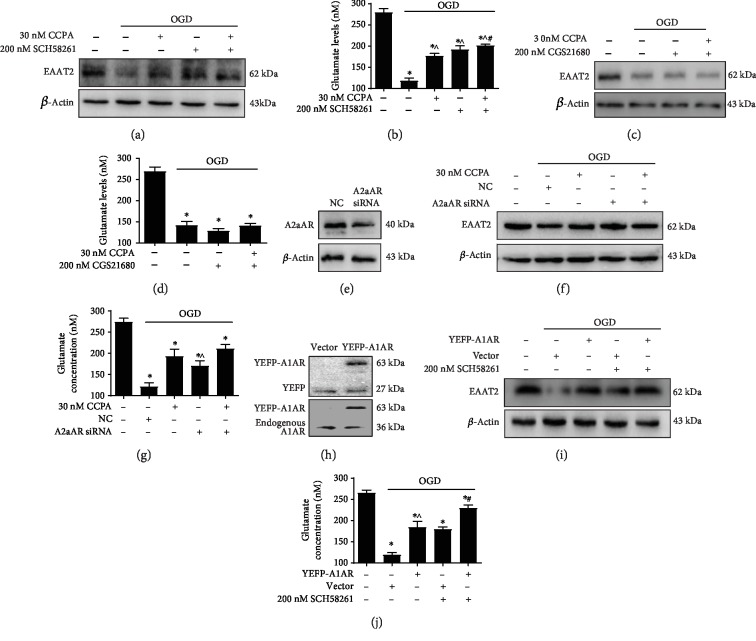
The A1AR/A2aAR heterodimer regulated the expression of EAAT2 and glutamate uptake. Astrocytes were subjected to OGD conditions in the absence or presence of 30 nM CCPA (an A1AR agonist) and 200 nM SCH58261 (an A2aAR antagonist). (a, b) The expression of EAAT2 and the intracellular glutamate level were detected by western blotting or with a glutamate assay kit, respectively. The data represent as the means ± S.E. (*n* = 3). ^∗^*p* < 0.05 vs. the control group; ^^^*p* < 0.05 vs. the OGD group; ^#^*p* < 0.05 vs. the OGD/CCPA group. Astrocytes were subjected to OGD conditions in the absence or presence of 30 nM CCPA and 200 nM CGS21680 (an A2aAR agonist). (c, d) The expression of EAAT2 and the intracellular glutamate level were detected by western blotting or with a glutamate assay kit, respectively. The data represent as the means ± S.E. (*n* = 3). ^∗^*p* < 0.05 vs. the control group. (e) The protein levels of A2aARafter the transfection of A2aAR siRNA into astrocytes for 48 h were evaluated by western blotting. A2aAR-silenced astrocytes were subjected to OGD conditions in the absence or presence of 30 nM CCPA. (f, g) The expression of EAAT2 and the intracellular glutamate level were detected by western blotting or with a glutamate assay kit, respectively. The data represent as the means ± S.E. (*n* = 3). ^∗^*p* < 0.05 vs. the control group and ^∧^*p* < 0.05 vs. the OGD group. (h) The protein level of A1AR after transfection of the EYFP-A1AR plasmid into astrocytes for 48 h was evaluated by western blotting. Astrocytes were transfected with the EYFP-A1AR plasmid for 48 h and then subjected to OGD conditions in the absence or presence of 200 nM SCH58261. (i, j) The expression of EAAT2 and the intracellular glutamate level were detected by western blotting or with a glutamate assay kit, respectively. The data represent as the means ± S.E. (*n* = 3). ^∗^*p* < 0.05 vs. the control group; ^^^*p* < 0.05 vs. the OGD group; ^#^*p* < 0.05 vs. the OGD/EYFP-A1AR group.

We explored the role of A1AR and A2aAR in the regulation of EAAT2 and glutamate uptake. The activation of A1AR with CCPA and the inactivation of A2aAR with SCH58261 elevated EAAT2 protein levels in astrocytes under OGD conditions (Figure 2(a)). Similarly, compared to those in the group under OGD alone, intracellular glutamate levels were increased in the OGD/CCPA, OGD/SCH58261, and OGD/CCPA+SCH58261 groups (*p* < 0.05). Interestingly, the level of glutamate was higher in the OGD/CCPA+SCH58261 group than that in the OGD/CCPA group (*p* < 0.05) (Figure 2(b)). Furthermore, the expression of EAAT2 and the level of glutamate were not significantly increased in the OGD/CGS21680+CCPA group compared to the group under OGD alone (*p* > 0.05) (Figures 2(c) and 2(d)). These data indicate that A1AR activation and A2aAR inactivation can elevate the expression of EAAT2 and improve glutamate uptake in astrocytes under OGD conditions. To further confirm the role of A1AR and A2aAR in the regulation of EAAT2 expression, A2aAR was knocked down with A2aAR siRNA (Figure 2(e)). The levels of EAAT2 protein and intracellular glutamate were increased in A2aAR-silenced cells under OGD compared to cells were under OGD alone (*p* < 0.05) (Figures 2(f) and 2(g)). Similarly, the forced expression of A1AR by the EYFP-A1AR plasmid partly reversed the OGD-mediated downregulation of EAAT2 expression and impaired glutamate uptake (Figures 2(h)–2(j)). These data suggested that A2aAR interacts with A1AR and forms an A1AR/A2aAR heterodimer, resulting in the suppressive activity of A1AR and glutamate uptake through the inhibition of EAAT2 expression.

### 3.3. PPAR*γ* Regulated Glutamate Uptake by EAAT2

**Figure 3:**
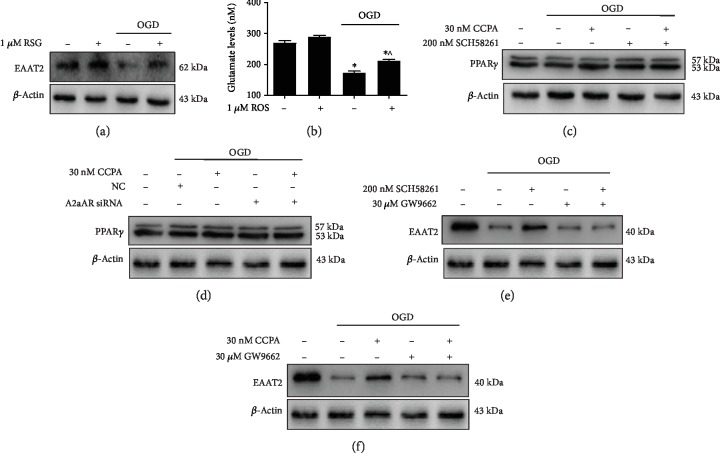
PPAR*γ* regulated the expression of EAAT2 and glutamate release from astrocytes. (a, b) EAAT2 protein and intracellular glutamate levels in astrocytes under OGD in the absence or presence of 1 *μ*M rosiglitazone were detected by western blotting or with a glutamate assay kit, respectively. The data represent the means ± S.E. (*n* = 3). ^∗^*p* < 0.05 vs. the control group and ^^^*p* < 0.05 vs. the OGD group. (c) Western blot analysis of the expression of PPAR*γ* in astrocytes treated with 30 nM CCPA and 200 nM SCH58261 that were exposed to OGD for 6 h. D. The PPAR*γ* protein level in astrocytes transfected with A2aAR siRNA for 48 h before being subjected to OGD conditions with 30 nM CCPA was evaluated. (e, f) Western blot analysis of the expression of EAAT2 in astrocytes treated with 30 nM CCPA or 200 nM SCH58261 in the presence of 30 *μ*M GW9662, followed by exposure to OGD for 6 h.

We then evaluated whether PPAR*γ* regulates the expression of EAAT2 and glutamate uptake. Astrocytes were pretreated with rosiglitazone (RSG), a PPAR*γ* agonist and subjected to OGD. PPAR*γ* activation increased the expression of EAAT2 and the intracellular glutamate level compared to those in the group under OGD alone (Figures 3(a) and 3(b)). These data suggested that PPAR*γ* can promote the expression of EAAT2 and attenuate OGD-impaired glutamate uptake. We further studied whether the A1AR/A2aAR heterodimer regulates PPAR*γ* expression.  Figure 3(c) shows that the expression of PPAR*γ* increased when astrocytes were preincubated with CCPA and SCH58261 and then treated with OGD compared to astrocytes treated with OGD alone. A2aAR silencing elevated the level of PPAR*γ* in cultures under OGD in the absence or presence of CCPA (Figure 3(d)). Additionally, GW9962, an inhibitor of PPAR*γ*, abrogated the CCPA- and SCH58261-induced increase in EAAT2 expression under OGD conditions (Figures 3(e) and 3(f)). These results suggest that the A1AR/A2aAR heterodimer regulates EAAT2 levels through PPAR*γ*.

### 3.4. The A1AR/A2aAR Heterodimer Regulated the Expression of YY1

**Figure 4:**
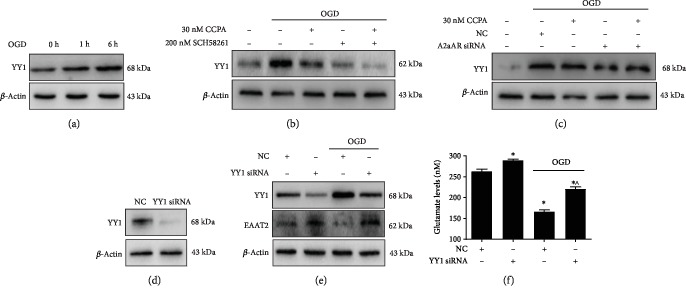
The A1AR/A2aAR heterodimer regulated EAAT2 expression via YY1. (a) The YY1 protein level in astrocytes under OGD conditions for 1 h and 6 h was evaluated. (b) Western blot analysis of YY1 expression in cells treated with 30 nM CCPA and 200 nM SCH58261 that were exposed to OGD for 6 h. (c) The YY1 protein level in astrocytes transfected with A2aAr siRNA for 48 h, followed by exposure to OGD conditions with 30 nM CCPA, was evaluated. The expression of PPAR*γ* and YY1 in treated astrocytes was evaluated by western blotting. (d) The YY1 protein after the transfection of YY1 siRNA into astrocytes for 48 h was evaluated by western blotting. YY1-silenced astrocytes were subjected to OGD conditions in the absence or presence of 30 nM CCPA. (e, f) The expression of YY1 and EAAT2 and the intracellular glutamate level were detected by western blotting or with a glutamate assay kit, respectively. The data represent the means ± S.E. (*n* = 3). ^∗^*p* < 0.05 vs. the NC group and ^^^*p* < 0.05 vs. the OGD/NC group.

In this study, we found that expression of the YY1 protein, a transcription factor, was increased (Figure 4(a)) after astrocytes were placed under OGD conditions. Further study showed that the expression of YY1 decreased when astrocytes were preincubated with CCPA and SCH58261 and then placed under OGD conditions compared to OGD alone (Figure 4(b)). Moreover, the expression of YY1 in A2aAR-silenced cultures under OGD in the absence or presence of CCPA was attenuated (Figure 4(c)). These data suggested that the A1AR/A2aAR heterodimer regulates YY1 expression.  Figure 4(d) shows that YY1 siRNA knocked down YY1 protein levels. YY1 silencing promoted the expression of EAAT2 in cells under both normal and OGD conditions (Figure 4(e)). The glutamate uptake assay showed that YY1 silencing increased intracellular glutamate levels in cells under OGD conditions (Figure 4(f)), suggesting that YY1 silencing can attenuate OGD-impaired glutamate uptake.

### 3.5. HDAC1 Recruitment by YY1 Repressed PPAR*γ*

**Figure 5:**
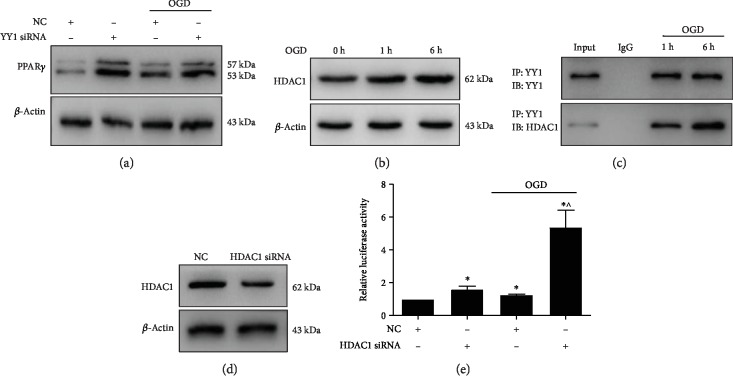
YY1 regulated PPAR*γ* by HDAC1. (a) The PPAR*γ* protein level in astrocytes transfected with YY1 siRNA for 48 h and then exposed to OGD for 6 h was evaluated. (b) The protein level of HDAC1 in astrocytes exposed to OGD for 6 h was measured. (c). Co-IP assays were used to evaluate the interaction between YY1 and HDAC1. (d) The protein level of HDAC1 in astrocytes transfected with HDAC1 siRNA for 48 h was evaluated by western blotting. (e) Luciferase assays evaluated the PPAR*γ* promoter activity in 293 T cells transfected with HDAC siRNA for 48 h and then exposed to OGD for 6 h. The data represent as the means ± S.E. (*n* = 3). ^∗^*p* < 0.05 vs. the NC group and ^^^*p* < 0.05 vs. the NC/OGD group.

The above results revealed that YY1 and PPAR*γ* can regulate the expression of EAAT2; however, the relationship between YY1 and PPAR*γ* is unknown. We found that YY1 silencing elevated the level of PPAR*γ* in cells under both normal and OGD conditions (Figure 5(a)). Moreover, the expression of HDAC1 was increased after OGD challenge (Figure 5(b)). Further study showed that OGD promoted the interaction between YY1 and HDAC1 (Figure 5(c)), suggesting that YY1 can recruit HDAC1 under OGD conditions. A luciferase assay showed that HDAC1 silencing significantly promoted PPAR*γ* promoter activity (Figures 5(d) and 5(e)). These data suggest that YY1 recruits HDAC1 to inhibit PPAR*γ* promoter activity under OGD conditions, resulting in the repression of EAAT2 expression and glutamate uptake in astrocytes.

## 4. Discussion

Stress induced the formation of homo-oligomeric and hetero-oligomeric GPCR complexes and it might interrupt the function of A1AR or A2aAR [31]. Herein, we found that OGD promoted the formation of A1AR-A2aAR heteromers and the activation of A1AR or inactivation of A2aAR attenuated OGD-mediated EAAT2 inhibition and glutamate uptake dysfunction. PPAR*γ* could regulate EAAT2-mediated glutamate uptake [43]. Therefore, we investigated whether A1AR-A2aAR heteromers regulated EAAT2 through PPAR*γ*. These data revealed that A1AR/A2aAR heterodimer by activation of A1AR or inactivation of A2aAR attenuated OGD-mediated suppress PPAR*γ* suppression, resulting in elevating the level of intracellular glutamate. Next, we further study the mechanism of A1AR-A2aAR heteromers to control EAAT2 via PPAR*γ*. Given YY1 could repress the expression of EAAT2 [21] and was a transcription factor for regulation target gene, such as PPAR*γ* [44]. We carried out experiments and revealed that YY1 suppressed the expression of EAAT2 through the recruitment of HDAC1 to the PPAR*γ* promoter region. These data suggested A1AR-A2aAR heterodimerization regulation of EAAT2 expression and glutamate uptake through YY1-induced recruitment of HDAC1 to the PPAR*γ* promoter region.

GPCRs, such as A1AR and A2aAR, detect and transmit extracellular chemicals into cells and activate downstream signals, resulting in the initiation of physiological and pathological responses [45]. In general, GPCRs are monomeric receptors that are present and function at the cell surface [46]. However, recent studies revealed the presence of homo-oligomeric and hetero-oligomeric GPCR complexes at the surface of cultured cells [47, 48]. These homo/heterodimers might be regulated in various ways, such as through agonists/antagonists. In particular, the functions of GPCR heterodimers, including their pharmacological properties and activation, may be significantly different than those of GPCR monomers [49, 50]. Several studies showed that A2aAR and A1AR interact and heterodimerize in hippocampal and cortical synaptosomes [28, 29, 51]. Additionally, A2aAR in this heterodimer can decrease the affinity of A1AR for its agonist [28]. In the present study, OGD promoted the interaction of A1AR with A2aAR in astrocytes and suppressed the expression of EAAT2 and glutamate uptake. A previous study showed that the activation of A2aAR also impaired glutamate clearance [33] and that the activation of A1AR played a protective role in ischemia [34, 35, 52]. Therefore, we suspected that OGD induces A1AR-A2aAR heterodimerization and that A2aAR activation suppresses A1AR-mediated regulation of EAAT2 expression. Further study demonstrated that both A1AR activation and A2aAR inactivation improved the OGD-mediated decrease in EAAT2 protein levels and damage to glutamate uptake. Interestingly, A2aAR agonist preconditioning blocked A1AR activation-induced changes in EAAT2 expression and glutamate uptake. These data prove that OGD mediates A1AR-A2aAR heterodimerization and suppresses the activity of A1AR, resulting in impaired glutamate uptake through the inhibition of EAAT2 expression.

PPAR*γ*, a member of the nuclear receptor superfamily of ligand-activated transcription factors, regulates gene transcription by binding peroxisome proliferator response elements (PPREs) in the promoter regions of target genes [53]. Activation of PPAR*γ* plays a critical role in immunity, inflammation [54], and metabolism [55]. Recently, Tureyen et al. showed that the activation of PPAR*γ* by RSG suppressed the inflammatory response and decreased the infarct volume and neurological deficits in middle cerebral artery occlusion (MCAO) model rats [56]. Ching and colleagues showed that the levels of extracellular glutamate were controlled by PPAR*γ* activity in both U87MG and U251MG cells [57]. Another study demonstrated that PPAR*γ* has a neuroprotective effect by upregulating EAAT2 promoter activity in astrocytes [43]. Consistent with previous reports, PPAR*γ* was able to promote the expression of EAAT2 and attenuate OGD-impaired glutamate uptake. We found that the activation of A1AR and the inactivation of A2aAR could increase the level of PPAR*γ* in astrocytes exposed to OGD conditions. These data suggested that A1AR/A2aAR heteromers control EAAT2 levels and glutamate uptake through regulating PPAR*γ* expression.

YY1, a transcription factor, regulates multiple proteins involved in cell proliferation and differentiation, apoptosis, and chromatin remodeling in various cell types [58–63]. Recent research has revealed that YY1 plays a critical role in neural development, neuronal function, developmental myelination, and neurological diseases [64]. Aguirre et al. and Rosas et al. found that YY1 represses GluT (GLAST)/EAAT1 expression through binding its promoter in chick glial cells [65, 66]. Further study also demonstrated that YY1 negatively regulates EAAT1 and EAAT2 expression in rat astrocytes [21, 67]. Consistent with the above documents, in our study, OGD induced YY1 expression, and YY1 silencing elevated EAAT2 protein levels and attenuated OGD-impaired glutamate uptake in mice astrocytes. Interestingly, the expression of YY1 was suppressed through the activation of A1AR or the inactivation of A2AR. These data suggested that OGD-mediated A1AR/A2aAR heteromers upregulate YY1 expression, following which YY1 represses EAAT2 expression and impairs glutamate clearance.

Previous studies have reported that YY1 regulates numerous genes at the transcriptional level [68]. The negative regulatory effect of YY1 was found to be dependent on HDACs, a class of histone deacetylases [69, 70]. Our results show that OGD induced the expression of HDAC1, which interacted with YY1. Romera et al. showed that the suCEBPD/HDAC1/HDAC3 complex inactivated PPARG2 transcription during adipocyte-like lipogenesis in HepG2 cells [43]. Herein, we also found that YY1 or HDAC1 silencing elevated PPAR*γ* levels in cells under both normal and OGD conditions and that HDAC1 knockdown could inhibit PPAR*γ* promoter activity under OGD conditions. These data suggest that YY1 recruits HDAC1 to inhibit PPAR*γ* promoter activity under OGD conditions.

**Figure 6:**
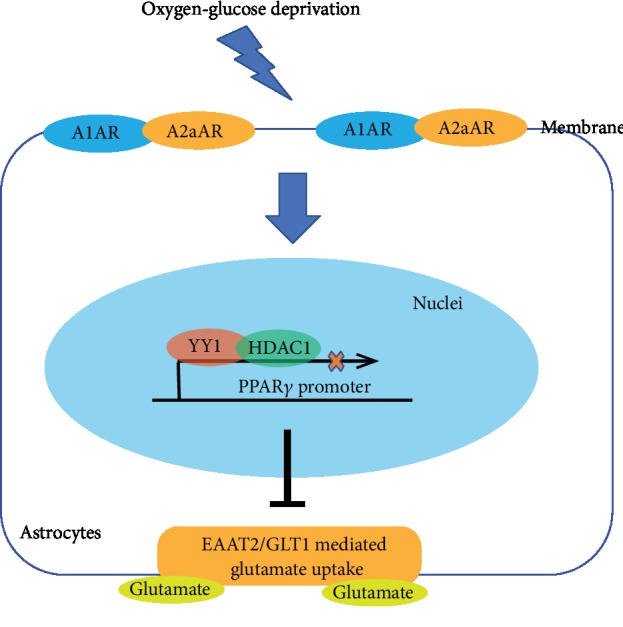
Schematic illustration of adenosine receptor A1-A2a heteromers regulates PPAR*γ* transcription and EAAT2 expression in astrocytes. OGD promoted A1AR-A2aAR heteromer formation and upregulated YY1 expression to suppress the PPAR*γ* transcription through recruitment of HDAC1 to the PPAR*γ* promoter region, resulting in abolished EAAT2-mediated glutamate uptake.

Taken together, these results suggest a mechanistic model for the A1AR-A2aAR heterodimerization-mediated regulation of EAAT2 expression and glutamate uptake through YY1-induced recruitment of HDAC1 to the PPAR*γ* promoter region. In this model, OGD promotes A1AR-A2aAR heterodimer formation. This complex elevates YY1 protein levels, represses EAAT2 expression, and impairs the uptake of glutamate by astrocytes. These effects can be partly reversed by the activation of A1AR or the inactivation of A2aAR. Additionally, YY1 suppresses the expression of EAAT2 through the recruitment of HDAC1 to the PPAR*γ* promoter region (Figure 6). These findings give insight into the mechanism by which multiple ARs regulate EAAT2 and should facilitate the development of therapeutics for ischemic stroke.

## Data Availability

The data used to support the findings of this study are available from the corresponding author upon request.

## Supplementary Materials

Supplementary Materials The supplement file showed the information on materials and reagents, including brand and lot numbers.
