# Supplementary material for: Adenosine Receptor A1-A2a Heteromers Regulate EAAT2 Expression and Glutamate Uptake via YY1-Induced Repression of PPARγ Transcription
Source: PPAR Res. 2020 Mar 6;2020:2410264. doi: 10.1155/2020/2410264 (PMC7079221; doi:10.1155/2020/2410264)
Supplement: Supplementary Materials — The supplement file showed the information on materials and reagents, including brand and lot numbers. [file 2410264.f1.docx]

Adenosine receptor A1-A2a heteromers regulate EAAT2 expression and glutamate uptake via YY1-induced repression of PPARγ transcription

Xianhua Hou^1^, Yuan Li^1^, Yuanyuan Huang^1^, Huan Zhang^1^, Li Gui^1*^.

^1^Department of Neurology, Southwest Hospital, Third Military Medical University (Army Medical University), Chongqing 400038, China

*Correspondence:

Email: guiligl_7519@sina.com

Tel.: +86 23 68 765263

**Meterial list**

| DMEM (High Glucose) | GIBCO | Lot: #10569044 |
| --- | --- | --- |
| DMEM (No, Glucose) | GIBCO | Lot: #A1443001 |
| Opti-MEM medium | GIBCO | Lot: #31985070 |
| Fetal bovine serum | GIBCO | Lot: #10099141 |
| Trypsin-EDTA (0.25%) | GIBCO | Lot: #25200072 |
| Adenosine A1 Receptor antibody | Abcam | Lot: #ab82477  WB: 1:1000, over night 4℃ |
| Adenosine Receptor A2a antibody | Abcam | Lot: # ab3461  WB: 1:500, over night 4℃  IP:1:50, over night 4℃ |
| GFAP antibody [EP672Y] | Abcam | Lot: #ab33922  IF: 1:200, over night 4℃ |
| S100 beta antibody [EP1576Y] | Abcam | Lot: # ab52642  IF: 1:200, over night 4℃ |
| PPARγ antibody [EPR18516] | Abcam | Lot: #ab178860  WB: 1:500, over night 4℃ |
| EAAT2 Antibody | CST | Lot: # 3838  WB: 1:1000, over night 4℃ |
| α/β-Tubulin Antibody | CST | Lot: #2148  WB: 1:1000, over night 4℃ |
| β-Actin (8H10D10) Antibody | CST | Lot: #3700  WB: 1:1000, over night 4℃ |
| YY1 (D5D9Z) Rabbit mAb | CST | Lot: #46395  WB: 1:500, over night 4℃  IP:1:50, over night 4℃ |
| HDAC1 | CST | Lot:# 34589  WB: 1:500, over night 4℃ |
| Glutamate Assay Kit | Abcam | Lot: # ab83389 |
| Protein A/G PLUS-Agarose | Santa Cruz | Lot: #sc-2003 |
| 2-Chloro-N6-cyclopentyladenosine (CCPA) | Abcam | Lot: # ab120434 |
| SCH58261 | Selleck | Lot: # S8104 |
| CGS21680 | Selleck | Lot: #2153 |
| HRP-Goat Anti-Rabbit IgG (H+L) | JACKSON | Lot:# 111-035-045  WB: 1:10000, RT 2h |
| HRP-Goat Anti-Mouse IgG (H+L) | JACKSON | Lot:# 111-035-003  WB: 1:10000, RT 2h |
| Alexa Fluor® 488-Goat Anti-Rabbit IgG (H+L) | JACKSON | Lot:# 111-545-003  IF: 1:200, over night 4℃ |
| Alexa Fluor® 594-Goat Anti-Rabbit IgG (H+L) | JACKSON | Lot:# 111-585-003  IF: 1:200, over night 4℃ |
| TRIzol reagent | SIGMA | Lot:#T9242 |
| GoTaq® qPCR Master Mix | Promega | Lot:#A6001 |
| GoScript™ RT Mix | Promega | Lot:#A6001 |
| Dual-Luciferase® Reporter Assay System | Promega | Lot:#E1910 |
| FuGENE® HD Transfection Reagent | Promega | Lot:#E2311 |
| T-PER™ Tissue Protein Extraction Reagent | Thermo fisher | Lot:#78510 |
| Mem-PER^TM^ PLUS Kit | Thermo fisher | Lot:#89842 |
| Polyvinylidene fluoride (PVDF) | GE | Lot:#A10161067 |
| Immobilon Western Chemiluminescence HRP Substrate (ECL kit) | Merck Millipore | Lot:# WBKLS0100 |
